# Reactogenicity Differences between Adjuvanted, Protein-Based and Messenger Ribonucleic Acid (mRNA)-Based COVID-19 Vaccines

**DOI:** 10.3390/vaccines12070802

**Published:** 2024-07-19

**Authors:** Matthew D. Rousculp, Kelly Hollis, Ryan Ziemiecki, Dawn Odom, Anthony M. Marchese, Mitra Montazeri, Shardul Odak, Laurin Jackson, Hadi Beyhaghi, Seth Toback

**Affiliations:** 1 Novavax, Inc., Gaithersburg, MD 20878, USA; amarchese@novavax.com (A.M.M.); mmontazeri@novavax.com (M.M.); hbeyhaghi@novavax.com (H.B.); stoback@novavax.com (S.T.); 2 RTI Health Solutions, Research Triangle Park, NC 27709, USA; khollis@rti.org (K.H.); rziemiecki@rti.org (R.Z.); dodom@rti.org (D.O.); sodak@rti.org (S.O.); gibson@rti.org (L.J.)

**Keywords:** booster, COVID-19, reactogenicity, real-world evidence, SARS-CoV-2, NVX-CoV2373, mRNA

## Abstract

Participants in studies investigating COVID-19 vaccines commonly report reactogenicity events, and concerns about side effects may lead to a reluctance to receive updated COVID-19 vaccinations. A real-world, post hoc analysis, observational 2019nCoV-406 study was conducted to examine reactogenicity within the first 2 days after vaccination with either a protein-based vaccine (NVX-CoV2373) or an mRNA vaccine (BNT162b2 or mRNA-1273) in individuals who previously completed a primary series. Propensity score adjustments were conducted to address potential confounding. The analysis included 1130 participants who received a booster dose of NVX-CoV2373 (*n* = 303) or an mRNA vaccine (*n* = 827) during the study period. Within the first 2 days after vaccination, solicited systemic reactogenicity events (adjusted) were reported in 60.5% of participants who received NVX-CoV2373 compared with 84.3% of participants who received an mRNA vaccine; moreover, 33.9% and 61.4%, respectively, reported ≥3 systemic reactogenicity symptoms. The adjusted mean (95% CI) number of systemic symptoms was 1.8 (1.6–2.0) and 3.2 (3.0–3.4), respectively. Local reactogenicity events (adjusted) were reported in 73.4% and 91.7% of participants who received NVX-CoV2373 and mRNA vaccines, respectively; the adjusted mean (95% CI) number of local symptoms was 1.5 (1.33–1.61) and 2.4 (2.31–2.52), respectively. These results support the use of adjuvanted, protein-based NVX-CoV2373 as an immunization option with lower reactogenicity than mRNAs.

## 1. Introduction

To date, multiple types of vaccines have been developed to protect against COVID-19. As the need for additional COVID-19 vaccination continues, messenger RNA (mRNA)- and protein-based vaccines are expected to be the most widely utilized platforms. The mRNA COVID-19 vaccines developed by Pfizer (BNT162b2) and Moderna (mRNA-1273) are approved for use in the United States (US) and Canada [1,2,3], and a protein-based vaccine formulated with saponin-based Matrix-M™ adjuvant developed by Novavax is authorized for use in the US and approved for use in Canada [3,4].

Participants in COVID-19 vaccine clinical trials have commonly reported mild and transient reactogenicity events within 7 days of receiving a vaccine, with many events resolving within 2 days post-vaccination [5,6,7,8,9,10,11]. These symptoms include local (injection-site) reactions (pain, tenderness, erythema, or swelling) or systemic reactions (fatigue, malaise, muscle pain, joint pain, nausea/vomiting, headache, or fever), which may increase with subsequent doses of a COVID-19 vaccine [6,9,10,11,12]. Local and systemic reactions are also commonly reported for immunizations targeting infectious diseases other than COVID-19 (e.g., influenza or shingles) [13,14]. Of note, studies comparing reactogenicity have generally found a higher incidence of local and systemic events following the receipt of mRNA COVID-19 vaccines compared with influenza and other non–COVID-19 vaccines, as well as a higher frequency of reactogenicity-associated medication use, sick leave, and doctor’s visits [15,16].

COVID-19-vaccine–related reactogenicity events can affect work and other daily activities, leading to absenteeism from work [17,18] and presenteeism [17,19], as well as vaccine hesitancy [20,21,22,23,24]. Indeed, concern over vaccine side effects was found to be the most common reason for refusing an updated COVID-19 vaccine [24]. The 2019nCoV-406 study surveyed participants in the US and Canada who were receiving a COVID-19 vaccine to compare how reactogenicity impacted work and other daily activities [17]. Findings suggest that in the 6 days following vaccination, recipients of the protein-based NVX-CoV2373 vaccine had lower unadjusted reactogenicity rates and had trended toward less overall impairment relative to recipients of mRNA vaccines (BNT162b2 and mRNA-1273). We also observed that over 90% of the most frequently occurring solicited reactogenicity events were reported within the first 2 days after vaccination. Here, we present an additional analysis of the 2019nCoV-406 study, including adjustment for potential confounding, to more closely examine reactogenicity within 2 days of receiving an authorized/approved COVID-19 vaccine in previously vaccinated participants.

## 2. Materials and Methods

### 2.1. Study Design and Participants

The prospective, noninterventional, observational 2019nCoV-406 study investigated the impact of common reactogenicity events from the COVID-19 vaccine on absenteeism, presenteeism, and work productivity loss [17]. Briefly, the study enrolled working adults aged 18 to <65 years in the US and Canada who voluntarily received an authorized/approved primary series or booster dose of a COVID-19 vaccine. As this was an observational study, participants selected the COVID-19 vaccine type they wanted to receive. A booster dose was defined as any COVID-19 dose received after the completion of a primary COVID-19 vaccination series, regardless of the prior vaccine type used or prior COVID-19 disease status. Booster vaccinations included NVX-CoV2373 (5 µg recombinant spike protein co-formulated with 50 µg of Matrix-M adjuvant) or an mRNA vaccine (BTN162b2 (30 μg) and mRNA-1273 (50 μg)). Participants completed baseline/screening questionnaires on the day of their vaccine (day 0) and a daily diary for the following 6 days that included a Vaccine Symptoms Diary. Further details on participant inclusion criteria and study methods are available in the prior publication [17]. Written informed consent was provided by each participant prior to the receipt of their requested vaccine dose and survey completion.

### 2.2. Objectives

The descriptive/comparative goal of this post hoc analysis was to determine the difference in local or systemic reactogenicity events occurring within the first 2 days after the receipt of an NVX-CoV2373 vaccine versus an mRNA COVID-19 vaccine, administered after completion of any primary vaccination series.

### 2.3. Assessments

The Vaccine Symptoms Diary measured 11 solicited, participant-reported local/systemic symptoms over a 24-h recall period. Solicited systemic reactogenicity symptoms were fever, fatigue, malaise, muscle pain, joint pain, nausea/vomiting, and headache. Solicited local reactogenicity symptoms included pain, tenderness, swelling, and redness at the injection site. All symptoms were recorded using a 0-to-3 response scale based on the worst level of reactogenicity severity experienced, with responses categorized as 0 (no symptom present), 1 (mild/no interference with activity), 2 (moderate/interferes with activity), and 3 (severe/significant interference with activity). A participant was considered to have experienced a reactogenicity symptom if they reported a severity of at least grade 1. This analysis used data from the 2 days immediately following vaccination.

### 2.4. Statistics

Analyses of reactogenicity in the 2 days following vaccination were completed in the booster dose population, which consisted of participants who received any booster dose of an approved/authorized COVID-19 vaccine, regardless of which vaccine type was used for prior doses or if they had previously had COVID-19. Due to low numbers receiving other vaccine types, only data related to NVX-CoV2373 or the mRNA vaccines (BNT162b2 and mRNA-1273) were included in this analysis. Estimation of the sample size required to power the primary objectives in the main study was described previously [17].

Results are presented by vaccine groups composed of participants who received NVX-CoV2373 and participants who received BNT162b2 or mRNA-1273, referred to as the mRNA vaccine group. Findings are also presented in subgroups for the different mRNA vaccines (BNT162b2 or mRNA-1273) and country (US or Canada).

Analyses are presented separately for systemic and local reactogenicity. The proportions of participants with any systemic/local reactogenicity and with individual systemic/local reactogenicity events, mean numbers of systemic/local events, and the proportion of participants with ≥3 systemic events are presented as comparative analyses. Comparative analyses were adjusted to address potential confounding using the inverse probability of treatment weighting (IPTW). As described previously [17], each participant was assigned a propensity score based on a select group of demographic and clinical characteristics identified using standardized differences. The present analysis used the following covariates in the IPTW model: country (US vs. Canada), prior COVID-19 diagnosis (yes vs. no), race (Asian, White), job category (professional), work at home (yes, no, or prefer not to answer), gender identity (male, female), and scheduled to work in the next 24 h (yes vs. no). The comparative analyses were weighted using the stabilized inverse probability of treatment weights. Due to the sample size limitation, the event severity and all subgroup analyses were analyzed descriptively. All results use the overall participant booster sample. Results were not adjusted for multiple comparisons.

## 3. Results

### 3.1. Participants

The 2019nCoV-406 study was conducted between July 2022 and March 2023. The booster population included 1130 participants, 303 of whom received NVX-CoV2373 and 827 who received an mRNA vaccine during the study period. Baseline demographics and clinical characteristics were generally balanced between vaccine groups, as reported previously [17]; however, some differences between the NVX-CoV2373 and mRNA vaccine groups were observed related to ethnicity (Table 1). A higher proportion of Hispanic, Latin American, or Latinx (50.8%) and White (50.2%) participants received NVX-CoV2373 versus an mRNA vaccine (25.0% and 33.6%, respectively). By contrast, more Asian (22.9%) and Native Hawaiian or Pacific Islander (9.1%) participants received an mRNA vaccine versus NVX-CoV2373 (13.2% and 2.0%, respectively). Medical conditions that put participants at high risk for severe COVID-19 were relatively low in both groups (NVX-CoV2373, 6.3%; mRNA, 5.4%). Of those participants who received NVX-CoV2373, more did so as a first (60.7%) versus second (39.3%) dose after completion of the primary series. Alternatively, of those who received an mRNA vaccine, more received this as a second (62.6%) versus first (37.4%) booster dose.

Of the 1130 participants in the booster population, 631 participants (NVX-CoV2373, *n* = 205; mRNA, *n* = 426) were from the US and 499 (NVX-CoV2373, *n* = 98; mRNA, *n* = 401) were from Canada (Table 1). The baseline demographics and characteristics of participants from the US and Canada were generally similar to those observed in the overall booster population. Most participants with a Hispanic, Latin American, or Latinx ethnicity came from the US, and most participants with an Asian or Native Hawaiian or Pacific Islander ethnicity came from Canada.

### 3.2. Systemic Reactogenicity

**Figure 1:**
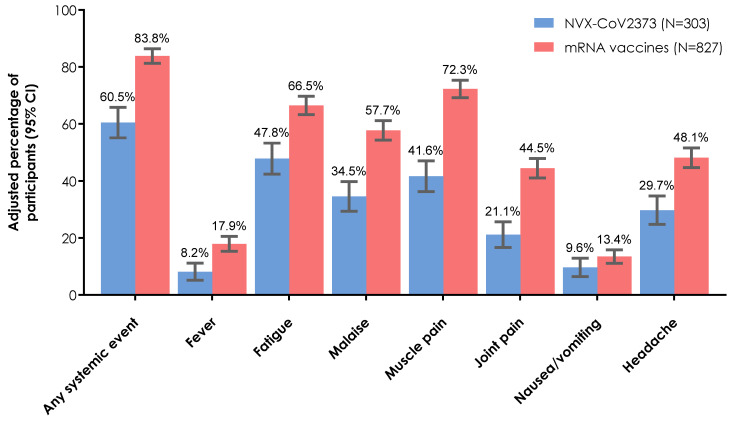
Rates of overall and individual solicited systemic reactogenicity events within 2 days of booster vaccination (IPTW adjusted estimates). Results are presented for the booster population. CI, confidence interval; IPTW, inverse probability of treatment weighting.

Within the first 2 days after vaccination, solicited systemic reactogenicity events were reported in 56.4% of participants who received NVX-CoV2373 and 84.4% of participants who received an mRNA vaccine (BNT162b2: 84.5%; mRNA-1273: 84.3%). After IPTW adjustment, 60.5% of participants who received the NVX-CoV2373 reported solicited systemic reactogenicity events compared with 83.8% of participants who received an mRNA vaccine (Figure 1). Muscle pain (NVX-CoV2373: 41.6%; mRNA vaccine: 72.3%), fatigue (47.8% and 66.5%, respectively), and malaise (34.5% and 57.7%, respectively) were the most common systemic events in each vaccine group.

**Figure 2:**
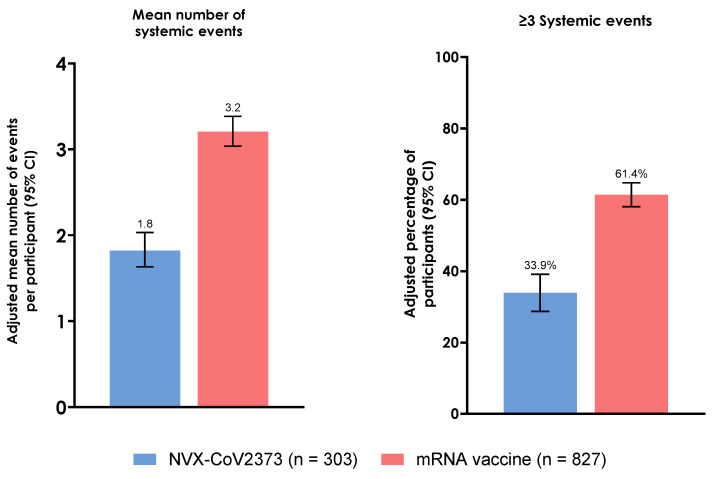
Summary of solicited systemic reactogenicity events within 2 days of booster vaccination (IPTW adjusted estimates). Booster population. CI, confidence interval; IPTW, inverse probability of treatment weighting.

Participants who received NVX-CoV2373 reported a mean number (SD) of 1.8 (2.0) systemic events, whereas those who received an mRNA vaccine reported a mean of 3.2 (2.1) systemic events (Table 2). Adjusting for confounding by IPTW led to similar results, with mean numbers of events (95% CI) of 1.8 (1.6–2.0) for the NVX-CoV2373 group and 3.2 (3.0–3.4) for the mRNA vaccine group (Figure 2). Markedly fewer participants who received NVX-CoV2373 reported three or more systemic reactogenicity events than those who received an mRNA vaccine (adjusted values (95% CI): NVX-CoV2373, 33.9% (28.7–39.1%); mRNA vaccine, 61.4% (58.1–64.8%)).

With respect to the event severity, a lower proportion (unadjusted) of participants who received NVX-CoV2373 reported a moderate or severe/significant systemic event within 2 days of vaccination compared with participants who received an mRNA vaccine (27.7% (84/303) vs. 49.3% (408/827)). Mild events were also reported in lower proportions of participants who received NVX-CoV2373 (28.7%) compared with those who received an mRNA vaccine (35.1%).

The mean (SD) number of systemic events was similar whether participants received BNT162b2 (3.0 (2.1)) or mRNA-1273 (3.5 (2.2)). Similarly, the proportion of participants reporting any systemic event was 84.5% (424/502) and 84.3% (274/325), respectively. However, a higher proportion of participants receiving mRNA-1273 reported three or more systemic reactogenicity events (unadjusted; 67.7% (220/325)) compared with BNT162b2 (57.6% (289/502)). The proportion of participants reporting moderate-to-severe systemic events was 51.7% (168/325) and 47.8% (240/502) with mRNA-1273 and BNT162b2, respectively (Table 2).

When assessed by country, unadjusted proportions of systemic reactogenicity events in the US were lower in participants who received NVX-CoV2373 (48.5%) compared with participants who received an mRNA vaccine (79.1%) (Table 3). Corresponding proportions tended to be higher for both vaccine types in Canadian participants (NVX-CoV2373: 72.5%; mRNA: 90.0%). Regardless of country, participants who received NVX-CoV2373 reported fewer systemic reactogenicity events (mean (SD): US, 1.6 (2.1); Canada, 2.1 (2.0)) than those who received an mRNA vaccine (mean (SD): US, 3.1 (2.3); Canada, 3.3 (1.9)). Similarly, regardless of country, fewer participants who received NVX-CoV2373 reported three or more events (US: 29.8% (61/205); Canada: 35.7% (35/98)) compared with those who received an mRNA vaccine (US: 58.2% (248/426); Canada: 64.8% (260/401)), and fewer participants who received NVXCoV2373 reported moderate-to-severe events (US: 22.0% (45/205)); Canada: 39.8% (39/98)) compared with those who received an mRNA vaccine (US: 45.5% (194/426); Canada: 53.4% (214/401)).

### 3.3. Local Reactogenicity

**Figure 3:**
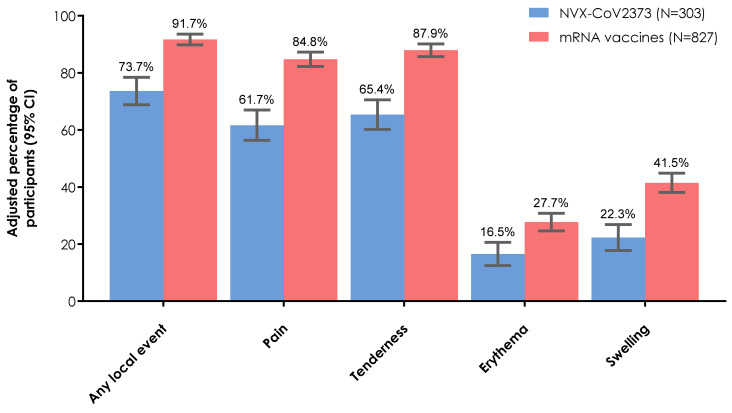
Overall and individual solicited local reactogenicity events within 2 days of booster vaccination (IPTW adjusted estimates). Booster population. CI, confidence interval; IPTW, inverse probability of treatment weighting.

Similar to systemic reactogenicity, a lower proportion of participants who received NVX-CoV2373 (68.3%) experienced at least one solicited local reactogenicity event, compared with those who received an mRNA vaccine (91.9%; BNT162b2, 92.6%; mRNA-1273, 90.8%). After IPTW adjustment, local reactogenicity events were estimated to be reported in 73.7% and 91.7% of participants who received a booster dose of NVX-CoV2373 and mRNA vaccine, respectively (Figure 3). This trend in differences continued, with the overall frequency of each individual event occurring in a higher proportion of participants in the mRNA vaccine versus NVX-CoV2373 group. For both the NVX-CoV2373 and mRNA vaccine groups, the most common solicited local events were pain (61.7% and 84.8%, respectively) and tenderness (65.4% and 87.9%, respectively) at the injection site.

The mean number (SD) of reported local reactogenicity events per individual was 1.5 (1.3) for participants who received NVX-CoV2373 and 2.4 (1.1) for those who received an mRNA vaccine. Mean (95% CI) adjusted numbers were 1.5 (1.3–1.6) and 2.4 (2.3–2.5), respectively. Compared with the NVX-CoV2373 group (21.1%), the proportion (unadjusted) of participants reporting moderate or severe/significant local reactogenicity events was 2.5-fold higher for participants in the mRNA vaccine group (52.0%) (Table 4).

Reporting of local events was generally similar whether participants received BNT162b2 or mRNA-1273 (Table 4). The proportion (unadjusted) of participants reporting any local event was 92.6% (465/502) for BNT162b2 and 90.8% (295/325) for mRNA-1273. The mean number (SD) of local events reported was similar between subgroups (2.3 (1.0) and 2.6 (1.2), respectively); however, the proportion of participants who reported moderate or severe local events was higher in recipients of mRNA-1273 (57.5% (187/325)) than recipients of BNT162b2 (48.4% (243/502)).

In both the US and Canada, proportions (unadjusted) of local reactogenicity events were lower in participants who received NVX-CoV2373 compared with participants who received an mRNA vaccine, with events reported at a lower frequency in US versus Canadian participants (US: NVX-CoV2373, 62.4% vs. mRNA, 88.3%; Canada: NVX-CoV2373, 80.6% vs. mRNA, 95.8%) (Table 5). The mean number of local reactogenicity events reported in the US and Canada tended to follow the same pattern as the overall booster population, with fewer events reported for those who received NVX-CoV2373 versus an mRNA vaccine. Similar to the overall population, regardless of country, lower proportions of participants who received NVX-CoV2373 reported moderate-to-severe local reactogenicity events (US: 20.0% (41/205); Canada: 23.5% (23/98)) compared with those who received an mRNA vaccine (US: 46.0% (196/426); Canada: 58.4% (234/401)).

## 4. Discussion

In this post hoc analysis of the 2019nCoV-406 study, participants who received NVX-CoV2373 after completing a primary vaccination series reported fewer and less-severe local and systemic reactogenicity symptoms in the 2 days following vaccination compared with those who received an mRNA vaccine after completing a primary vaccination series. These real-world data provide a better understanding of the reactogenicity events associated with the two COVID-19 vaccine platforms (protein-based and mRNA) received in the US and Canada. In the 2 days following vaccination, local and systemic reactogenicity events were more commonly reported in participants who received an mRNA vaccine than in those who received NVX-CoV2373. The proportions of participants reporting the most common local (injection site pain and tenderness) and systemic (muscle pain, fatigue, and malaise) reactogenicity events were higher among those who received an mRNA vaccine (>84% and >57%, respectively) than among those who received NVX-CoV2373 (up to 65% and up to 48%, respectively). In addition, mRNA vaccine recipients were more likely to report experiencing three or more systemic events or events with moderate-to-severe severity than participants who received NVX-CoV2373. These findings are consistent regardless of the mRNA brand compared.

These data build on overall findings from the 2019nCoV-406 study [17] by focusing on the 2 days immediately following the receipt of a booster vaccination and adjusting for potential confounding using IPTW. As noted previously, this timing is of interest because over 90% of the most frequently occurring solicited reactogenicity events in the 2019nCoV-406 study were reported within the first 2 days. Similarly, multiple studies have shown that systemic and local reactogenicity events are short-lived, with mean or median durations of 2 days or less for the most frequently reported solicited reactogenicity events [7,8,9,11]. The primary analysis from the 2019nCoV-406 study suggested a trend toward less overall work impairment with NVX-CoV2373 compared with the mRNA vaccines [17]. In addition, greater proportions of participants who received an mRNA vaccine reported local and systemic reactogenicity events compared with NVX-CoV2373. This post hoc analysis showed consistent findings for the 2 days after the booster vaccination, including when the potential confounding related to differences in baseline and clinical characteristics being accounted for using IPTW.

Multiple studies have found a greater reactogenicity of mRNA vaccines relative to other COVID-19 vaccine platforms, including adjuvanted, protein-based vaccines such as NVX-CoV2373 [12,16,25,26,27,28,29,30]. While there are limitations to direct comparisons between different studies, it is notable how closely these findings from the 2019nCoV-406 study mirror observations from the Oxford COV-BOOST trial and a National Institute for Allergy and Infectious Diseases and National Institutes of Health-funded booster study [26,27]. The results from this analysis of 2019nCoV-406 expand on previous reports [16,28,30] by focusing on the effects of the vaccine doses administered after the completion of the primary COVID-19 vaccination series. Additional COVID-19 vaccine dosing, whether as the second dose of the primary series or as subsequent doses, can result in greater reactogenicity compared with the first injection [6,9,10], especially when receiving a heterologous booster [19,25,31]. Although the studies investigating reactogenicity in homologous and heterologous booster doses were generally not powered to make comparisons between vaccine types, altogether findings from 2019nCoV-406 and other studies suggest that NVX-CoV2373, when administered as a heterologous booster, has less reactogenicity than mRNA vaccines when administered as a homologous or heterologous booster dose [12,25,26,30]. This is especially relevant as most people in the US and Canada have received a primary series of an mRNA vaccine. Concern about side effects is a primary reason for COVID-19 vaccine hesitancy [22,23,24]. Accordingly, COVID-19 vaccines with lower rates of reactogenicity, which is suggested of heterologous use of NVX-CoV2373 by the present analysis and several descriptive studies [12,25,26,30], have the potential to decrease vaccine hesitancy. This may be especially useful given the continued evolution of SARS-CoV-2 and recommendations from public health authorities for annual updates to the COVID-19 vaccine strain composition [32,33,34,35], suggesting the potential need for a seasonal COVID-19 vaccination, similar to influenza vaccination.

Comparison between the two mRNA vaccines showed that BNT162b2 (30 μg) tended to elicit fewer and slightly less severe systemic and local reactogenicity events than mRNA-1273 (50 μg). If making cross-study comparisons, it is important to note the vaccine doses investigated in the studies [12,25,26,27,28]. In particular, participants in COV-BOOST and the MixNMatch Study (DMID 21-0012) received a higher dose of mRNA-1273 (100 μg) than is currently approved [26,27]. However, mRNA-1273 was administered at the approved 50 μg dose [2] in the 2019nCoV-406 study, and the rates of the reactogenicity events observed with RNA-1273 were still higher than those observed with BNT162b2.

Despite the widespread use of mRNA and protein-based COVID-19 vaccines, the mechanisms that induce the immune responses associated with reactogenicity and immunogenicity remain largely unknown. However, studies using animal models suggest that the mRNA and protein-based vaccines induce the innate immune system through different pathways, driven by unique platform designs and mechanisms [36,37,38,39]. The mRNA COVID-19 vaccine components elicit intrinsic adjuvant-like effects due to the immunostimulatory activation of innate sensors by lipid nanoparticle (LNP) carriers and foreign nucleic acids, as well as by direct LNP host-cell cytotoxicity [36]. By contrast, the protein-based COVID-19 vaccine relies on the saponin-based Matrix-M adjuvant to enhance the immunogenicity of protein antigens. Following Matrix-M–adjuvanted vaccine administration, both saponins and antigen are undetectable within 24 h post-injection (hpi) having been transported to the draining lymph nodes, thereby seeing local cytokine expression declining sharply by 48 h [39]. Though some animal studies have found LNP-mRNA persists at the injection site beyond 24 hpi, head-to-head comparisons of mRNA and protein–vaccine trafficking kinetics, clearance, and proinflammatory cytokine expression are needed. Differences in reactogenicity could also be due to the high concentrations of mRNA (30–50 µg), which utilizes host-cell machinery to translate variable antigen quantities compared to the precise 5 µg of the antigen contained in the protein vaccine.

Recent studies have highlighted the significant protective properties of both mRNA and Novavax COVID-19 vaccines. The mRNA vaccines, such as those developed by Pfizer–BioNTech and Moderna, have demonstrated the high efficacy rates of approximately 94–95% in preventing symptomatic COVID-19 in clinical trials, with robust protection against severe disease and hospitalization [6,9]. Additionally, the mRNA vaccines have shown effectiveness in reducing transmission rates, contributing to community-level immunity [40,41]. Similarly, the Novavax vaccine, which utilizes a recombinant nanoparticle technology combined with the Matrix-M adjuvant, has shown an efficacy rate of approximately 90% in phase 3 clinical trials [7,8] and 100% against SARS-CoV-2–related hospitalization [42]. The Novavax vaccine also offers strong protection against SARS-CoV-2 variants of concern such as the Alpha and Beta variants, which underscores its potential as a critical tool in the ongoing global vaccination effort [43]. Together, these vaccines represent powerful options in the fight against COVID-19, offering high levels of protection. Limitations to the 2019nCoV-406 study have been previously reported [17] and are inherent to all noninterventional, real-world investigations. For example, study participants may not be representative of all populations receiving COVID-19 vaccines. This may be due to the timing of the study relative to vaccine approval/authorization; the later authorization for use and the availability of NVX-CoV2373 (winter/early spring 2023) relative to that of mRNA vaccines (late summer/early fall 2022) may have created a temporal bias. Low enrollment at some study sites likely led to the concentration of participants with particular demographics (e.g., race/ethnicity) or with access to a specific type of vaccine. In addition, while the availability of different vaccine types may have been defined by what was available at the site, vaccines were selected by the participant, which could also introduce bias. Real-word studies using patient-reported outcomes are often associated with concerns regarding the completeness and accuracy of patient reporting. While participants were instructed to complete the daily questions at the same time every day, it was possible to access and complete the diaries within an 8-h period.

It is relevant to note that the study was not powered to evaluate reactogenicity and that the post hoc nature of this analysis limits the conclusions that can be derived from the results. IPTW adjustments were made for the overall booster dose population; however, adjusted analyses were not available for data reporting on the severity or for the mRNA vaccine and country subgroups. Results in the country subgroups were largely similar to those of the overall population; however, some differences were observed. A higher proportion of participants in the US (32% (205/630)) selected NVX-CoV2373 compared with participants in Canada (20% (98/499)); reactogenicity tended to be reported in higher proportions of Canadian participants, regardless of the vaccine received, and the differences in reactogenicity reporting between NVX-CoV2373 and mRNA vaccine subgroups tended to be greater in participants in the US compared with those from Canada. Some of these findings may be limited by differences in adverse event reporting in the US and Canada; however, reactogenicity was higher among recipients of mRNA vaccines compared with NVX-CoV2373 in both countries. Finally, the impact of booster dose number (e.g., first vs. second booster dose) relative to reactogenicity was not assessed.

The 2019nCoV-406 study provides data from a large, real-world population of participants receiving an additional dose of COVID-19 vaccine after completion of a primary vaccination series. Reactogenicity was captured in the same manner as in the phase 3 studies of COVID-19 vaccines, thereby strengthening the generalizability of these findings. Importantly, participants were enrolled at the time of their vaccination, with data collected daily, improving accuracy and limiting recall bias. In addition, this post hoc analysis used propensity score adjustments (i.e., IPTW) to reduce bias.

## 5. Conclusions

Considering the lower frequency and intensity of COVID-19 reactogenicity symptoms observed in this post hoc analysis of the real-world 2019nCoV-406 study, this analysis supports the use of adjuvanted protein-based NVX-CoV2373 as an immunization option that has low reactogenicity. The benefits of traditional protein-based platforms are highlighted by a history of safe and effective use, as well as favorable storage and handling without the need for the ultra-cold temperatures required of mRNA, not to mention their inherent flexibility and the precise control of antigen concentration. Specifically, Matrix-M–adjuvanted NVX-CoV2373 has demonstrated consistent efficacy and immunogenicity, as well as evidence of lower reactogenicity than mRNA COVID-19 vaccine options. Future prospective studies are needed to confirm the observations reported here and to continue to examine the effects of age, sex, race/ethnicity, and even comorbidities on COVID-19 vaccine reactogenicity.

## Figures and Tables

**Table 1:** Baseline demographics and clinical characteristics of study participants by region.

	Booster Population [17]		US		Canada	
Parameter	NVX-CoV2373 (*n* = 303)	mRNA Vaccine ^a^ (*n* = 827)	NVX-CoV2373 (*n* = 205)	mRNA Vaccine ^a^ (*n* = 426)	NVX-CoV2373 (*n* = 98)	mRNA Vaccine ^a^ (*n* = 401)
**Age, mean (SD) years**	38.9 (11.8)	40.1 (13.0)	39.4 (12.0)	42.6 (13.4)	37.9 (11.2)	37.5 (12.0)
**Gender identity, *n* (%)**						
Female	156 (51.5)	469 (56.7)	109 (53.2)	243 (57.0)	47 (48.0)	226 (56.4)
Male	142 (46.9)	355 (42.9)	95 (46.3)	182 (42.7)	47 (48.0)	173 (43.1)
Genderfluid	1 (0.3)	0	0	0	1 (1.0)	0
Nonbinary	2 (0.7)	3 (0.4)	0	1 (0.2)	2 (2.0)	2 (0.5)
Prefer not to answer	2 (0.7)	0	1 (0.5)	0	1 (1.0)	0
**Race/ethnicity ^b^, *n* (%)**						
African American or Black	33 (10.9)	77 (9.3)	27 (13.2)	67 (15.7)	6 (6.1)	10 (2.5)
Asian ^c^	40 (13.2)	189 (22.9)	8 (3.9)	21 (4.9)	32 (32.7)	168 (41.9)
Hispanic, Latin American, or Latinx	154 (50.8)	207 (25.0)	145 (70.7)	197 (46.2)	9 (9.2)	10 (2.5)
Middle Eastern or North African ^d^	5 (1.7)	21 (2.5)	2 (1.0)	2 (0.5)	3 (3.1)	19 (4.7)
Native Hawaiian or Pacific Islander ^e^	6 (2.0)	75 (9.1)	2 (1.0)	0	4 (4.1)	75 (18.7)
White	152 (50.2)	278 (33.6)	106 (51.7)	157 (36.9)	46 (46.9)	121 (30.2)
Other ^f^	11 (3.6)	22 (2.7)	6 (2.9)	9 (2.1)	5 (5.1)	13 (3.2)
**Prior COVID-19 diagnosis, *n* (%)**	119 (39.3)	433 (52.4)	67 (32.7)	239 (56.1)	52 (53.1)	194 (48.4)
**Medical condition that puts participant at high risk for severe COVID-19 ^b^, *n* (%)**						
Diabetes	6 (31.6)	21 (46.7)	2 (1.0)	15 (3.5)	4 (4.1)	6 (1.5)
Hypertension	7 (36.8)	13 (28.9)	5 (2.4)	10 (2.3)	2 (2.0)	3 (7.5)
Heart disease	2 (10.5)	8 (17.8)	1 (6.7)	8 (1.9)	1 (1.0)	0
Respiratory conditions	5 (26.3)	11 (24.4)	4 (2.0)	7 (1.6)	1 (1.0)	4 (1.0)
Other	5 (26.3)	13 (28.9)	4 (2.0)	10 (2.3)	1 (1.0)	3 (0.7)
**Booster dose, *n* (%)**						
First	184 (60.7)	309 (37.4)	167 (81.5)	266 (62.4)	17 (17.3)	43 (10.7)
Second or later	119 (39.3)	518 (62.6)	38 (18.5)	160 (37.6)	81 (82.7)	358 (89.3)
**mRNA vaccine type, *n* (%)**						
Monovalent	-	652 (78.8)	-	401 (94.1)	-	251 (62.6)
Bivalent	-	175 (21.2)	-	25 (5.9)	-	150 (37.4)

Baseline demographics and clinical characteristics for the booster population have been published previously [17]. SD = standard deviation. ^a^ Individuals received either BNT162b2 or mRNA-1273. ^b^ The categories for these variables were not mutually exclusive (participants could have listed more than one). ^c^ Includes participants who identified as Chinese, South Asian (e.g., East Indian, Pakistani, or Sri Lankan), Southeast Asian (e.g., Vietnamese, Cambodian, Laotian, or Thai), Korean, or Japanese. ^d^ Includes participants who identified as Middle Eastern, North African, Arab, or West Asian (e.g., Iranian or Afghan). ^e^ Includes participants who identified as Native Hawaiian, Pacific Islander, or Filipino. ^f^ Race/ethnicity categories with fewer than 20 responses are captured in the “other” category and include: Alaska Native, American Indian, or Native American participants (total *n* = 6); race or ethnicity not listed (*n* = 16); and prefer not to answer (*n* = 11).

**Table 2:** Descriptive analysis of systemic reactogenicity events in the overall population and by mRNA vaccine subgroup (unadjusted).

	NVX-CoV2373 (*n* = 303)	mRNA Vaccine (*n* = 827)	mRNA Vaccine Subgroup	
			BNT162b2 (*n* = 502)	mRNA-1273 (*n* = 325)
Median (range)	1 (0–7)	3 (0–7)	3 (0–7)	4 (0–7)
Number of events, *n* (%)				
No systemic reactogenicity events	132 (43.6)	129 (15.6)	78 (15.5)	51 (15.7)
1	41 (13.5)	91 (11.0)	62 (12.4)	29 (8.9)
2	34 (11.2)	98 (11.9)	73 (14.5)	25 (7.7)
3	26 (8.6)	108 (13.1)	69 (13.7)	39 (12.0)
4	26 (8.6)	140 (16.9)	81 (16.1)	59 (18.2)
5	26 (8.6)	128 (15.5)	72 (14.3)	56 (17.2)
6	11 (3.6)	89 (10.8)	48 (9.6)	41 (12.6)
7	7 (2.3)	44 (5.3)	19 (3.8)	25 (7.7)
Severity, *n* (%)				
No reactogenicity symptoms reported	132 (43.6)	129 (15.6)	78 (15.5)	51 (15.7)
Mild/no interference with activities	87 (28.7)	290 (35.1)	184 (36.7)	106 (32.6)
Moderate/interfered with activities	62 (20.5)	288 (34.8)	177 (35.3)	111 (34.2)
Severe/significant interference with activities	22 (7.3)	120 (14.5)	63 (12.5)	57 (17.5)

Table shows descriptive data summarizing systemic reactogenicity events reported within 2 days of receipt of a booster vaccination in the booster population. All values are unadjusted.

**Table 3:** Descriptive analysis of systemic reactogenicity events by country (unadjusted).

	US		Canada	
	NVX-CoV2373 (*n* = 205)	mRNA Vaccine (*n* = 426)	NVX-CoV2373 (*n* = 98)	mRNA Vaccine (*n* = 401)
Mean (SD)	1.6 (2.1)	3.1 (2.3)	2.1 (2.0)	3.3 (1.9)
Median (range)	0 (0–7)	3 (0–7)	2 (0–7)	4 (0–7)
Any systemic event, *n* (%)	100 (48.5)	337 (79.1)	71 (72.5)	361 (90.0)
Number of events, *n* (%)				
No systemic reactogenicity events	105 (51.2)	89 (20.9)	27 (27.6)	40 (10.0)
1	23 (11.2)	41 (9.6)	18 (18.4)	50 (12.5)
2	16 (7.8)	48 (11.3)	18 (13.4)	50 (12.5)
3	18 (8.8)	52 (12.2)	8 (8.2)	56 (14.0)
4	12 (5.9)	57 (13.4)	14 (14.3)	83 (20.7)
5	20 (9.8)	63 (14.8)	6 (6.1)	65 (16.2)
6	6 (2.9)	45 (10.6)	5 (5.1)	44 (11.0)
7	5 (2.4)	31 (7.3)	2 (2.0)	12 (3.2)
Severity, *n* (%)				
No reactogenicity symptoms reported	105 (51.2)	89 (20.9)	27 (27.6)	40 (10.0)
Mild/no interference with activities	55 (26.8)	143 (33.6)	32 (32.7)	147 (36.7)
Moderate/interfered with activities	31 (15.1)	139 (32.5)	31 (31.6)	149 (37.2)
Severe/significant interference with activities	14 (6.8)	55 (12.9)	8 (8.2)	65 (16.2)

Table shows descriptive data summarizing systemic reactogenicity events reported within 2 days of receipt of a booster vaccination in the booster population. All values are unadjusted.

**Table 4:** Descriptive analysis of local reactogenicity events in the overall population and by mRNA vaccine subgroup (unadjusted).

	NVX-CoV2373 (*n* = 303)	mRNA Vaccine (*n* = 827)	mRNA Vaccine Subgroup	
			BNT162b2 (*n* = 502)	mRNA-1273 (*n* = 325)
Median (range)	2 (0–4)	2 (0–4)	2 (0–4)	3 (0–4)
Number of events, *n* (%)				
No systemic reactogenicity events	96 (31.7)	67 (8.1)	37 (7.4)	30 (9.2)
1	51 (16.8)	57 (6.9)	38 (7.6)	19 (5.8)
2	98 (32.3)	328 (39.7)	227 (45.2)	101 (31.1)
3	33 (10.9)	216 (26.1)	130 (25.9)	86 (26.5)
4	25 (8.3)	159 (19.2)	70 (13.9)	89 (27.4)
Severity, *n* (%)				
No reactogenicity symptoms reported	96 (31.7)	67 (8.1)	37 (7.4)	30 (9.2)
Mild/no interference with activities	143 (47.2)	330 (39.9)	222 (44.2)	108 (33.2)
Moderate/interfered with activities	57 (18.8)	338 (40.9)	200 (39.8)	138 (42.5)
Severe/significant interference with activities	7 (2.3)	92 (11.1)	43 (8.6)	49 (15.1)

Table shows descriptive data summarizing local reactogenicity events reported within 2 days of receipt of a booster vaccination in the booster population. All values are unadjusted.

**Table 5:** Descriptive analysis of local reactogenicity events by country (unadjusted).

	US		Canada	
	NVX-CoV2373 (*n* = 205)	mRNA Vaccine (*n* = 426)	NVX-CoV2373 (*n* = 98)	mRNA Vaccine (*n* = 401)
Mean (SD)	1.3 (1.3)	2.4 (1.3)	1.7 (1.2)	2.4 (1.0)
Median (range)	1 (0–4)	2 (0–4)	2 (0–4)	2 (0–4)
Any local event, *n* (%)	128 (62.4)	376 (88.3)	79 (80.6)	384 (95.8)
Number of events, *n* (%)				
No local reactogenicity events	77 (37.6)	50 (11.7)	19 (19.4)	17 (4.2)
1	33 (16.1)	36 (8.5)	18 (18.4)	21 (5.2)
2	59 (28.8)	133 (31.2)	39 (39.8)	195 (48.6)
3	20 (9.8)	108 (25.4)	12 (13.3)	108 (26.9)
4	16 (7.8)	99 (23.2)	9 (9.2)	60 (15.0)
Severity, *n* (%)				
No reactogenicity symptoms reported	77 (37.6)	50 (11.7)	19 (19.4)	17 (4.2)
Mild/no interference with activities	87 (42.4)	180 (42.3)	56 (57.1)	150 (37.4)
Moderate/interfered with activities	37 (18.1)	147 (34.5)	20 (20.4)	191 (47.6)
Severe/significant interferences with activities	4 (2.0)	49 (11.5)	3 (3.1)	43 (10.7)

Table shows descriptive data summarizing local reactogenicity events reported within 2 days of receipt of a booster vaccination in the booster population. All values are unadjusted.

## Data Availability

Data are available on request due to privacy restrictions. The data presented in this study are available in aggregate form on request to the corresponding author. The data are not publicly available due to privacy requirements.
